# Re-Emergence and Spread of Haemorrhagic Septicaemia in Germany: The Wolf as a Vector?

**DOI:** 10.3390/microorganisms9091999

**Published:** 2021-09-21

**Authors:** Peter Kutzer, Claudia A. Szentiks, Sabine Bock, Guido Fritsch, Tibor Magyar, Christoph Schulze, Torsten Semmler, Christa Ewers

**Affiliations:** 1 Landeslabor Berlin-Brandenburg, 15236 Frankfurt (Oder), Germany; sabine.bock@landeslabor-bbb.de (S.B.); christoph.schulze@landeslabor-bbb.de (C.S.); 2 Department of Wildlife Diseases, Leibniz Institute for Zoo and Wildlife Research, 10315 Berlin, Germany; szentiks@izw-berlin.de; 3 Department of Reproduction Management, Leibniz Institute for Zoo and Wildlife Research, 10315 Berlin, Germany; fritsch@izw-berlin.de; 4 Veterinary Medical Research Institute, Eötvös Lóránd Research Network (ELKH), 1143 Budapest, Hungary; magyar.tibor@vmri.hu; 5 Microbial Genomics, Robert Koch Institute, 13353 Berlin, Germany; SemmlerT@rki.de; 6 Faculty of Veterinary Medicine, Institute for Hygiene and Infectious Diseases of Animals, Justus Liebig University Giessen, 35392 Giessen, Germany; christa.ewers@vetmed.uni-giessen.de

**Keywords:** *Pasteurella multocida*, wild boar, domestic animals, core genome, MLST, virulence

## Abstract

Since 2010, outbreaks of haemorrhagic septicaemia (HS) caused by *Pasteurella* (*P*.) *multocida* capsular type B (*Pm*B) emerged in Germany. In 2017, we noticed a close spatiotemporal relationship between HS outbreak sites and wolf (*Canis lupus*) territories. Thus, the main objectives of our study were to investigate the molecular epidemiology of German *Pm*B-HS-isolates and to assess the role of wolves as putative vectors of this pathogen. We collected 83 *Pm*B isolates from HS outbreaks that occurred between 2010 and 2019 and sampled 150 wolves, which were found dead in the years 2017 to 2019, revealing another three *Pm*B isolates. A maximum-likelihood-based phylogeny of the core genomes of 65 *Pm*B-HS-isolates and the three *Pm*B-wolf-isolates showed high relatedness. Furthermore, all belonged to capsular:LPS:MLST genotype B:L2:ST122^RIRDC^ and showed highly similar virulence gene profiles, but clustered separately from 35 global ST122^RIRDC^ strains. Our data revealed that German HS outbreaks were caused by a distinct genomic lineage of *Pm*B-ST122 strains, hinting towards an independent, ongoing epidemiologic event. We demonstrated for the first time, that carnivores, i.e., wolves, might harbour *Pm*B as a part of their oropharyngeal microbiota. Furthermore, the results of our study imply that wolves can carry the pathogen over long distances, indicating a major role of that animal species in the ongoing epidemiological event of HS in Germany.

## 1. Introduction

Haemorrhagic septicaemia (HS) is an OIE listed disease of cattle and buffaloes characterised by an acute, highly fatal septicaemia with high morbidity and mortality, causing major economic losses especially in Asian and African countries [1]. Besides domestic and wild ruminants [2,3,4,5,6,7], domestic pigs and wild boar might also be affected [7,8,9,10,11]. 

HS is caused by the Gram-negative, facultative anaerobic bacterium *Pasteurella* (*P*.) *multocida*. Based on serological or DNA-based typing methods *P. multocida* isolates can be classified into 5 capsular types (A, B, D, E and F) [12], 16 serotypes (serovars 1 through 16) [13,14] and 8 LPS genotypes (L1 to L8) [15]. HS cases in Asia and Europe are commonly associated with serovar B:2, while serovar E:2 isolates have so far been limited to outbreaks in Africa. Two MLST schemes are available for *P. multocida* typing, namely the RIRDC and the multi-host scheme, both hosted at the *P. multocida* MLST website (https://pubmlst.org/pmultocida/). HS isolates have mainly been associated with sequence type ST122^RIRDC^ [7,16,17,18].

Apart from concurrent infections, poor nutrition or immunosuppression, high temperature and humidity are common stressors associated with HS outbreaks [7,19]. Therefore, the disease usually emerges in tropical areas in Africa, Asia, and the Middle East [20]. In Europe, HS cases have been reported in several countries, including Denmark, Hungary, Poland, and Spain during the last three decades [2,4,9,11,21,22]. In Germany, HS re-emerged in 2010 after its last noted occurrence in 1986 [7,18,23,24].

To date, the epidemiology of HS in Germany faces several open questions. In particular, the source of reintroduction of the infectious agent and its vehicle of spread over long distances are not known. Towards the end of 2017, we noted that HS outbreak sites might correspond to the habitats of wolves (*Canis lupus*) in Germany. This prompted us to investigate (i) the spatiotemporal relationship between known HS outbreak sites and established wolf territories; (ii) if wolves could harbour *P. multocida* capsular type B (*Pm*B) in their nasal and/or oropharyngeal microbiota and, if so; (iii) the relatedness of these isolates with HS outbreak isolates in terms of clonal relationship and possession of virulence associated genes. In addition, to clarify their position in the worldwide epidemiology of HS, the genomes of German *Pm*B isolates were compared to those of selected global HS-related isolates.

## 2. Materials and Methods

### 2.1. Bacterial Isolates

Between 2010 and 2019, we collected 83 *P. multocida* isolates from HS cases involving cattle (*Bos taurus*/*n* = 48), domestic pigs (*Sus scrofa domesticus*/*n* = 11) including one Göttingen minipig, European wild boars (*Sus scrofa scrofa*/*n* = 19), fallow deer (*Dama dama*/*n* = 3), a red deer (*Cervus elaphus*) and a horse (*Equus ferus caballus*) (Supplementary Table S1). The isolates from 2010 were already included in a previous publication [7]. In addition, three isolates from HS cases in cattle (Pm241 (IHIT37773)) and pigs (P56 (IHIT37770), P57 (IHIT37771)) in Hungary [11,22] and five global HS-related *Pm*B strains (5018, 5022, BF, M-1404, and NCTC 10323; Supplementary Table S2) provided by the Institute of Microbiology and Epizootics, Freie Universität Berlin, Germany, were used for comparison purposes.

Diseased animals showed various clinical signs of severe septicaemia, including dyspnoea, anorexia, ataxia, fever, and inflammatory swellings in the submandibular region, throat, and brisket. In domestic pigs, distinct cyanosis of the throat and brisket was frequently present. Necropsy findings included haemorrhagic diathesis, phlegmonous inflammation in the Waldeyer’s tonsillar ring and the throat, acute fibrinous pleuropneumonia, and splenomegaly. On Columbia blood agar and/or *Pasteurella* selective agar (both Thermo Fisher Scientific, Darmstadt, Germany), *P. multocida* was isolated from all tissues including bone marrow, mainly in heavy growth and, depending on the grade of decay, pure culture. All isolates were subjected to both the *P. multocida* multiplex capsular PCR [12] and the HS-causing type-B-specific PCR assay [10].

In a second step, between September 2017 and September 2019 bacterial swab samples from 150 wolves (*Canis lupus*) were collected (Supplementary Table S3). The animals were found dead predominantly as a consequence of traffic accidents (approx. 86.7%), but also due to poaching (6.7%) and infectious as well as non-infectious diseases (6.6%). Discovery sites were spread over ten German states. Specimens were taken during necropsy by separate swabbing of nose, palate, and tonsillar crypts. Swabs were immediately transferred to Amies transport medium (MAST Diagnostica, Reinfeld, Germany), sent to the laboratory, plated onto *Pasteurella* selective agar, and incubated (36 °C, 18–20 h, ambient air). Colonies resembling *P. multocida* were subcultured and identified using MALDI-TOF MS (MALDI Biotyper, Bruker, Bremen, Germany). *P. multocida* isolates were subjected to capsular and HS-specific PCR as stated above.

### 2.2. Spatiotemporal Analysis of HS Outbreaks

Spatiotemporal data of HS outbreaks, including at least the postal code, municipality, and date, were obtained from senders’ investigation orders or by personal request. Locations and designations of confirmed wolf territories per monitoring year (May of a given year to April of subsequent year) were collected from the “Federal Documentation and Consultation Centre on Wolves (DBBW)” database [25]. Georeferencing of HS outbreak sites and wolf territories but also distance measurement between a given HS outbreak site and the closest known wolf territory at that time was based on Google Maps (https://www.google.de/maps, accessed on 30 August 2021). For map visualisation, the QGIS 3.16.0 package [26] was used.

### 2.3. Whole Genome Sequencing (WGS)

Seventy-three *Pm*B isolates were whole genome sequenced in this study. These comprised of 65 *Pm*B isolates from Germany (Supplementary Table S1), representing various geographical regions and hosts, 3 isolates from Hungary and 5 recent HS-related *Pm*B strains from Asia and the U.S.A. (5018, 5022, BF, M-1404, and NCTC 10323). DNA was prepared using the DNeasy Blood & Tissue Kit (QIAGEN, Hilden, Germany). Sequencing was performed using the Illumina TrueSeq library preparation kit in accordance with the manufacturer’s recommendations (Illumina Inc., San Diego, CA, USA) and 250 bp paired-end sequencing was performed on the Illumina HiSeq 2000 (Illumina Inc.) platform with an average coverage of 100×. The Illumina paired-end reads were adapter-trimmed by Flexbar v.3.0.3 [27] and the draft de novo assembly was performed using SPAdes v.3.11.1 [28] with default settings. All genomes were annotated by Prokka v.1.13 [29].

### 2.4. Multilocus Sequence Typing and Phylogenetic Analysis

Multilocus sequence types of all isolates were initially determined by amplification and sequence analysis (Eurofins Genomics, Ebersberg, Germany) of the seven housekeeping genes used in the RIRDC-MLST scheme as previously described [7]. From the 73 whole genome sequenced isolates, multilocus sequence types (STs) were confirmed by using MLST 2.0 hosted by the Center for Genomic Epidemiology (https://cge.cbs.dtu.dk/services/MLST/, accessed on 9 August 2021). Isolates were assigned to STs according to the RIRDC (^RIRDC^) (seven-allele scheme using genes *adk*, *est*, *pmi*, *zwf*, *mdh*, *gdh*, and *pgi*) and the multi-host (^mh^) MLST scheme (seven-allele scheme using genes *adk*, *aroA*, *deoD*, *gdhA*, *g6pd*, *mdh*, and *pgi*) (https://pubmlst.org/organisms/pasteurella-multocida/, accessed on 9 August 2021). Open reading frames (ORFs) of the *P. multocida* genomes were predicted by Prokka v.1.13 [29] and were used as input for Roary v.3.12.0 [30] to calculate the core and accessory genome with a sequence identity threshold of 95% for similarity. Core genes were individually aligned with Mafft v.7.407 [31] and subsequently concatenated. This core gene alignment was used as input for the calculation of a maximum likelihood-based phylogeny with RAxML v.8.2.10 [32]. To compare recent global HS isolates with our isolates, 185 publicly available *P. multocida* genomes were downloaded from the NCBI website (https://www.ncbi.nlm.nih.gov/genome, accessed on 9 August 2021) and screened for ST122^RIRDC^, which represents the most frequently ST associated with *Pm*B-HS-strains.

### 2.5. Determination of Virulence-Associated Genes

WGS data were used to determine the presence of known virulence-associated genes (VAGs) among *P. multocida* isolates. Screening for VAGs was carried out by NCBI BLASTn (https://blast.ncbi.nlm.nih.gov/Blast.cgi, accessed on 9 August 2021) analysis against homologous genes present in a database of VAGs or virulence gene loci containing several genes, based on in-house created and manually curated VAG reference sequences (Supplementary Table S4). Coverage length and sequence identity thresholds were 80%/80%. We searched for genes belonging to the categories (i) adhesion and colonization (*ptfA*, *fimA*, *hsf-1*, *hsf-2*, *pfhB-igB, pfhB1, pfhB2,* and the non-specific tight adherence protein locus *flp1-flp2-tadV-rcpCAB-tadZABCDEFG*); (ii) iron regulation and iron acquisition (*afuCBA*, *ccmABCDEF*, *exbB*, *exbD*, *fecABDC*, *fbpABC*, *fur*, *hgbA*, *hgbB*, *tbpA*, and *tonB*); (iii) extracellular enzymes, such as those involved in sialic acid synthesis and catabolism (*nanB*, *nanH, neuA, nanATEK,* *nanR*, and *siaPT-nanM*), hyaluronidase (*pmHAS*), and superoxide dismutase (*sodA* and *sodC*); (iv) toxins (*toxA*); (v) type II and type III secretion systems (locus_tags “DR93_1687” and “DR93_1692”); and (vi) a variety of outer membrane proteins (*ompA*, *ompH*, *oma87*, and *plpB*). In addition, we screened the genomes of all isolates for genes encoding biosynthesis of capsule types A (*hyaD-hyaC*), B (*bdbD*), D (*dcbF*), E (*ecbJ*), and F (*fcbD*), and for lipopolysaccharide biosynthesis genes encoding for *P. multocida* LPS genotypes 1 to 8 (locus tags “NCTC10382_00021”, “NCTC10323_00016”, and “ATO47_06115”, genes *latB*, *rmlC*, *nctB*, *ppgB*, and *natF*) (Supplementary Table S4).

## 3. Results

### 3.1. Determination of P. multocida Capsular Type and HS Specific DNA Fragment KTT72/KTSP61

All 83 *P. multocida* isolates collected from HS cases in Germany tested positive for capsular type B synthesis gene *bdbD* by multiplex PCR. Out of the 150 wolves sampled, *P. multocida* was isolated from 29 animals (19.3%). Based on capsular type multiplex PCR, 2 isolates (1.3%) were assigned to capsular type A and 3 (2.0%) to capsular type B, while the remaining 24 isolates (16.0%) were negative for the capsule biosynthesis genes tested. *Pm*B from wolves were isolated from either palatine or tonsillar swabs, or both (Supplementary Table S3). All 86 *Pm*B isolates obtained from HS outbreaks and wolves harboured the HS-specific DNA fragment KTT72/KTSP61.

### 3.2. Spatiotemporal Analysis of HS Outbreaks in Respect of Proven Wolf Territories

**Figure 1:**
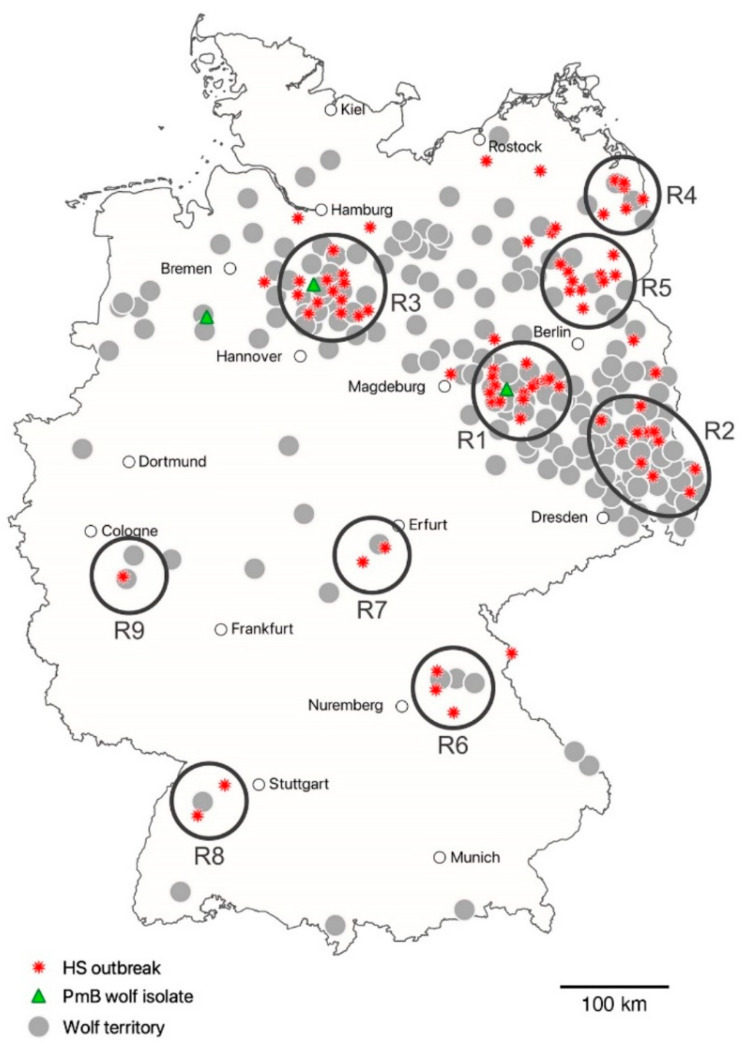
Location of 83 German HS outbreak sites (2010–2019) in relation to proven wolf territories. Regional outbreak clusters R1–R9 are indicated by black circles.

Out of the 83 HS outbreak sites, 70 could be assigned to 1 of 9 delimited and partially widely spaced regional clusters (R1–R9) (Supplementary Table S1, Figure 1). Except R5, all clusters had in common that the initial HS outbreak occurred after wolves established a territory there. In brief, the monitoring year of the first proven wolf territory versus the year of the first known HS outbreak was as follows: R1, 2008–2009 vs. 2010; R2, 2002–2003 vs. 2011; R3, 2011–2012 vs. 2013; R4, 2007–2008 vs. 2014; R5, 2017–2018 vs. 2014; R6, 2016–2017 vs. 2017; R7, 2014–2015 vs. 2018; R8, 2018–2019 vs. 2019; and R9, 2018–2019 vs. 2019 (Supplementary Figure S1). Out of the 83 HS outbreak sites, 13 were categorized as N/A, i.e., not assigned to one of the clusters R1 to R9.

Considering the official DBBW data, 25, 50, 75 and 90% of the HS cases investigated happened after wolves became resident in a territory with its assumed geographic centre 7, 10, 24, and 44 km linear distance away from the outbreak site, respectively. Only nine outbreaks occurred more than 40 km away from the closest wolf territory. The three *Pm*B isolates from wolves were assigned to R1 (IHIT36478, 2017), R3 (IHIT36485, 2018), and a region approx. 75 km west of R3 (IHIT36466, 2018) (Figure 1).

### 3.3. Multilocus Sequence Typing and Core Genome Comparison

The 86 HS-related isolates from Germany (*n* = 83) and Hungary (*n* = 3) and the 3 *Pm*B isolates from wolves belonged to sequence type ST122^RIRDC^ according to the RIRDC MLST scheme. Sixty-eight *Pm*B isolates from Germany (*n* = 65) and Hungary (*n* = 3) were whole genome sequenced. Following the multi-host (^mh^) MLST scheme for *P. multocida*, 58 isolates were assigned to ST64^mh^ (*adk*-26, *aroA*-28, *deoD*-23, *g6pd*-23, *gdhA*-6, *mdh*-22, *pgi*-25). Another ten isolates obtained from cattle (*n* = 4), domestic pigs (*n* = 3), but also from a wolf, a European wild boar, and a mini pig, belonged to ST61^mh^ (*adk*-26, *aroA*-29, *deoD*-23, *g6pd*-23, *gdhA*-6, *mdh*-22, *pgi*-25), a single locus variant of ST64^mh^.

To classify our *Pm*B isolates from HS cases and wolves into the global context of well-characterized HS reference and field strains, we performed a core genome comparison using publicly available genomes. As of June 2021, in silico screening of 185 *P. multocida* genomes retrieved from the NCBI website revealed the presence of 33 non-duplicate ST122^RIRDC^ strains from buffalo, cattle, bison, horse, and chicken, mostly from Asian countries (Bangladesh, China, India, Iran, Kazakhstan, Malaysia, Pakistan, and Thailand), but also from the U.S.A., and Scotland. Furthermore, we included another five *Pm*B strains, namely BF (IHIT37643, Asia), 5018 (IHIT37640, Asia), 5022 (IHIT37641, Asia), M-1404 (IHIT37651, U.S.A.), and NCTC 10323 (Myanmar) that were sequenced in this study and were confirmed as ST122^RIRDC^ (Supplementary Table S2). Three of the publicly available genomes (*Pm*B BUKK (JQAO01.1), *Pm*B Anand1_buffalo (ALBX01.1) and *Pm*B Anand1_cattle (ALBY01.1)) were excluded from core genome analysis as they clustered far outside from the other strains. The remaining 35 non-German and non-Hungarian *Pm*B strains mainly (82.9%) belonged to ST44^mh^ (*adk*-26, *aroA*-27, *deoD*-23, *g6pd*-23, *gdhA*-22, *mdh*-22, *pgi*-25), which represents a double locus variant of ST61^mh^ and ST64^mh^. Five isolates were determined as ST64^mh^ and one isolate from a domestic pig in China (HN04) was assigned as ST73-like^mh^ (*adk*-26, *aroA*-28, *deoD*-23, *g6pd*-23, *gdhA*-22, *mdh*-22-like (new), *pgi*-25).

**Figure 2:**
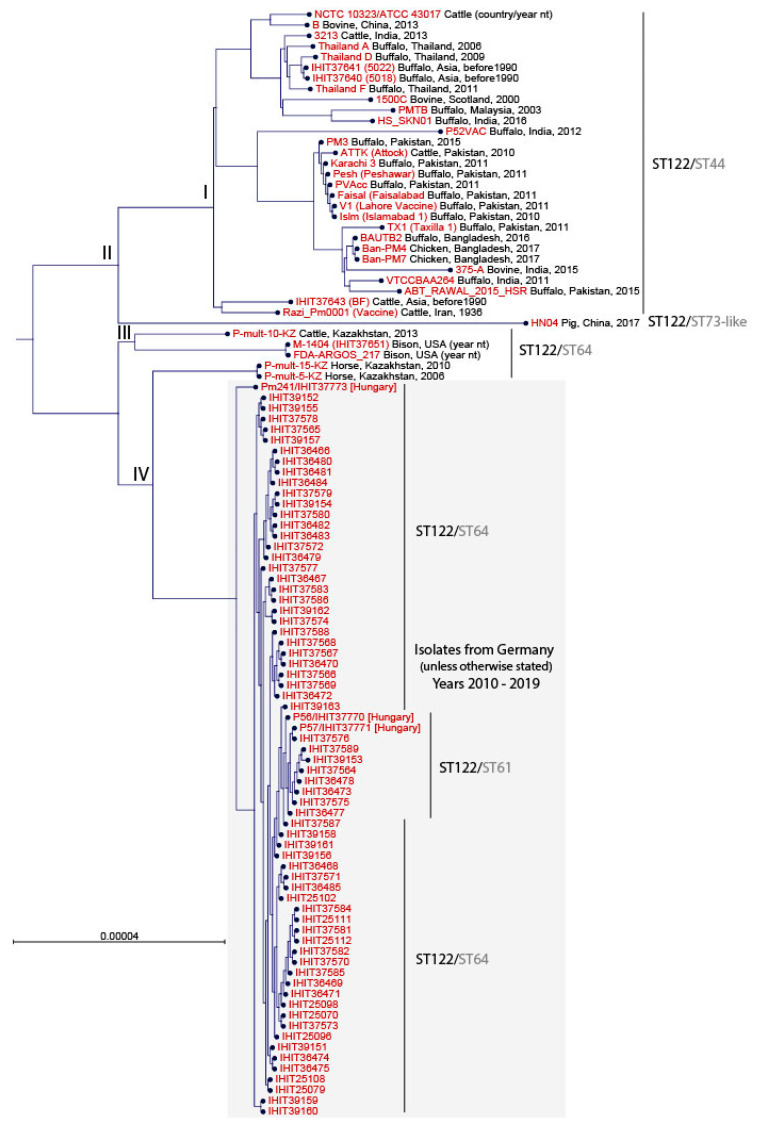
Neighbour-joining phylogenetic tree constructed from the core genome (1806 orthologous genes) of 103 *Pm*B isolates. Multilocus sequence types (ST^RIRDC^/ST^mh^) are indicated on the right, next to the isolate identifier.

A neighbour-joining phylogenetic tree based on the comparison of 1804 orthologous genes revealed that the German and Hungarian *Pm*B isolates clustered separately from strains from HS cases in different Asian countries, the U.S.A., and Scotland (Figure 2). Principally, the strains were grouped following their multi-host MLST types. According to their general clustering, we differentiated four major groups (I–IV). The first group (I) included 29 global strains of ST44^mh^. The porcine ST73-like^mh^ strain clustered separately (group II) and the third group (III) included three ST64^mh^ strains from Kazakhstan and the United States. Group IV contained all 68 ST64^mh^ and ST61^mh^ *Pm*B isolates from Germany and Hungary, and separated on another branch two ST64^mh^ strains from horses isolated in Kazakhstan in the years 2006 and 2010 (P-mult-5-KZ, P-mult-15-KZ). Within group IV, all German and the two Hungarian isolates P56 (IHIT37770) and P57 (IHIT37771) differed by 0 to 6 SNPs in 1804 genes. For the third isolate from Hungary, Pm241 (IHIT37773), we could determine 15 to 18 SNPs difference to the other isolates of our study. The closest similarity between global strains and isolates from our study was determined for two *Pm*B strains from horses (P-mult-5-KZ and P-mult-15-KZ) from Kazakhstan (67–71 SNPs).

**Figure 3:**
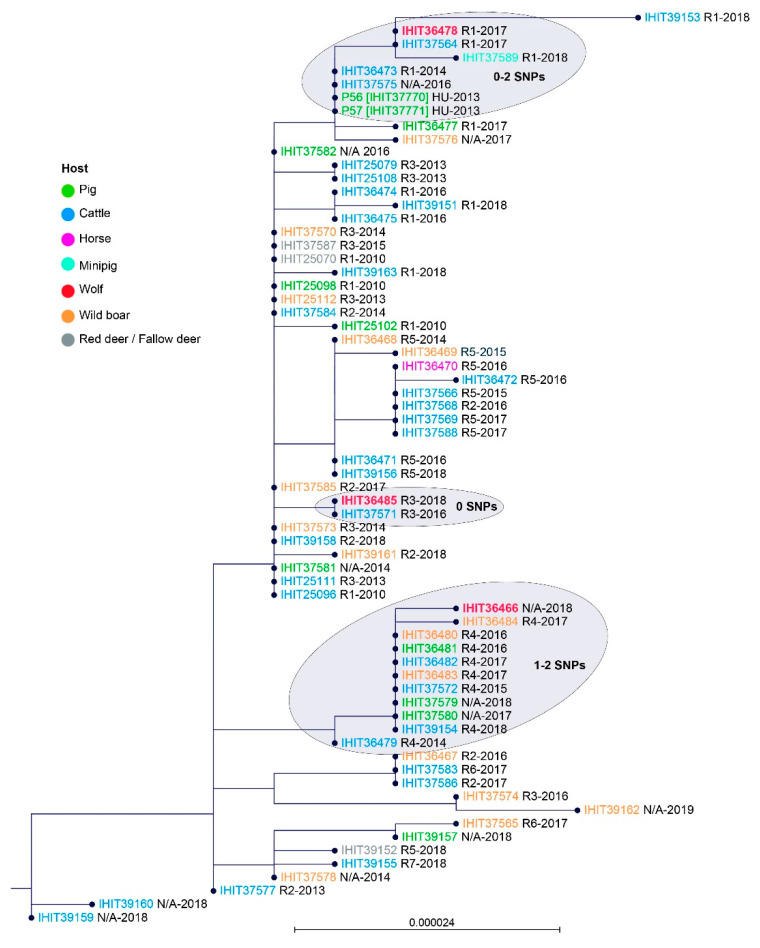
Neighbour-joining phylogenetic tree constructed from the core genome (2075 orthologous genes) of 67 *Pm*B isolates, namely 62 from German HS outbreaks, three from wolves (indicated in red and bold), and two from HS outbreaks among domestic pigs in Hungary. The host origin of *Pm*B isolates is colour-coded as indicated in the Figure legend. Geographic origin of isolates (R1–R9, N/A) and year of isolation are provided next to the isolate identifier. Clusters comprising genetically highly similar isolates from both wolves and domestic animals are shaded in grey (number of SNPs are indicated).

To further study the genetic relatedness of the isolates from wolves and other domestic and wild animals from Germany (and two very closely clustering isolates from Hungary), we performed a second comparison of the core genome of these 67 *Pm*B isolates. Based on a core set of 2075 genes, maximum SNP difference within all but one of the German isolates ranged from 0 to 24 SNPs. In addition, the two isolates from domestic pigs in Hungary (P56 (IHIT37770) and P57 (IHIT37771)) fell into this range. We identified several isolates that shared 100% in nucleotide sequences of their core genome (Figure 3). For example, in R5, isolates from five cattle (2015–2017) and one horse (2016) showed zero SNPs. Another group of genetically identical isolates comprised of three cattle, a domestic pig, and two European wild boars from R4 between 2015 and 2018, as well as two isolates from geographically more distant outbreaks (N/A) in domestic pigs in 2017 and 2018. The two Hungarian pig isolates, recovered in 2013, showed zero SNP difference to the German isolates IHIT36473 (cattle, R1) and IHIT37575 (cattle, R1) that were obtained in 2014 and 2016, respectively. Since a retrospective survey revealed no direct or indirect relations between the Hungarian pig farms and German outbreak sites, possible epidemiological links remain unsolved to date. Finally, another group of 13 isolates with identical core genomes included four animal species (domestic pig, European wild boar, fallow deer, and cattle) and was obtained from three different regional clusters (R1, R2, R3) but also two separated outbreak sites (N/A) between the years 2010 and 2018. The most separated isolate was IHIT39153 which was recovered from cattle in Germany in R1 in 2018, showing 28 to 43 SNPs difference in comparison to all other isolates, as depicted in Figure 3.

### 3.4. Distribution of Capsule, LPS, and Virulence-Associated Genes among PmB-ST122 Strains

Based on the analysis of WGS data of 68 *Pm*B isolates from wolves (*n* = 3), cattle (*n* = 33), European wild boars (*n* = 16), domestic pigs (*n* = 11), fallow deers (*n* = 2), a horse, a mini pig, and a red deer, the presence of capsular type B biosynthesis genes and the HS-specific DNA fragment KTT72/KTSP61 was confirmed. 

All 68 isolates as well as the 35 global strains harboured the capsular:LPS:MLST genotype B:L2:ST122^RIRDC^ that was previously associated with bovine HS [33]. A number of VAGs, including (i) adhesin genes *pfhB2*, *ptfA*, *fimA*, and *hsf*-2; (ii) iron-related genes or gene clusters, such as *afuCBA*, *fbpABC*, *exbBD-tonB*, *hgbB*, and others (Table 1); (iii) genes for extracellular enzymes like *nanH*, *neuA*, and *siPT-nanM*; (iv) outer membrane protein genes *ompA*, *ompH*, *oma87*, and *plpB*; and finally (v) genes *sodA* and *sodC*, that are related to bacterial oxidative stress response, were almost regularly present in the 68 European and 35 global HS strains (Table 1 and Supplementary Table S2). On the other hand, some VAGs, including autotransporter adhesin gene *hsf*-1, filamentous hemagglutinin 1 gene *pfhB1*, a locus encoding a non-specific tight-adherence protein, haemoglobin binding protein gene *hgbA*, regulatory protein *nanR*, hyaluronan synthase gene pmHAS, *P. multocida* toxin (PMT) gene *toxA*, and genes previously linked with type 2 and type 3 secretion systems were completely or nearly completely (one isolate positive for neuraminidase gene *nanB*) absent from all genomes screened. In conclusion, irrespective of their host (wolf, wild animal, or domestic animal) and geographical background the 103 *Pm*B-HS-genomes had almost identical VAG profiles. Global strains and German/Hungarian isolates merely differed significantly in the presence of transferrin binding protein A gene *tbpA*, which was present in 77.1% (all from bovines) of the 35 global strains but in none of the German/Hungarian isolates.

## 4. Discussion

The last official reports about cases of haemorrhagic septicaemia in Germany date back to 1986 [24]. However, several HS outbreaks in the past decade have confirmed the re-emergence of the disease in Germany [7,18]. Our data of 83 HS outbreak sites that were identified between 2010 and 2019 again provide clear evidence of an ongoing dissemination of *Pm*B strains not only in domestic pigs and cattle, but also in wild animals, such as European wild boar, fallow deer, red deer, and most notably, wolves, in different geographical regions of Germany. Previous publications have speculated about the primary source of HS outbreaks and about the way the pathogen could spread both over long distances and between different domestic and wild animal species [7,9,18]. Until now, infected or colonized wild animals such as fallow or roe deer (*Capreolus capreolus*) were considered possible carriers of *Pm*B [7].

In our study, bacteriological examination of nasal and oropharyngeal swabs from 150 wolves resulted in three (2.0%) *Pm*B isolates harbouring the HS-specific DNA fragment *ktt72/ktsp61*. However, it must be noted that the sensitivity of the detection method is questionable. Selectivity of the medium used is focused on, but not limited to, *Pasteurellaceae* in general. Therefore, *Pasteurella canis*, *Pasteurella dagmatis*, and *Frederiksenia canicola* (former Bisgaard taxon 16), overall representing up to 10% of the cultivable oral microflora of domestic dogs [34], were regularly found during this study. Furthermore, *P. multocida* non-typable by the multiplex capsular PCR, *P. multocida* capsular type A but also *Acinetobacter* spp., *Myroides* spp., *Neisseria animaloris*, *Pseudomonas* spp. and Gram-positive cocci grew in high abundance frequently. Finally, some animals showed a high degree of decay or were kept frozen until necropsy. Considering all these factors, *Pm*B might be underdiagnosed due to overgrowth by the accompanying flora or by just picking the wrong colonies.

All three *Pm*B-HS-isolates from wolves revealed a molecular signature that was frequently associated with bovine HS strains, namely the capsular:LPS:MLST genotype B:L2:ST122^RIRDC^ [17]. Notably, this genotype was also detected in all non-bovine *Pm*B-HS-genomes from our study, which is in accordance with previous findings, where the isolate HN04 that was recovered from swine in China, revealed this genotype as well [35]. As recently noted, it appears that the B:L2:ST122^RIRDC^ type shows a rather limited correlation with the corresponding host, but a high linkage with disease manifestation, i.e., HS [17]. The shared B:L2:ST122 ^RIRDC^ genotype among the genomes of three wolf isolates and HS isolates from Germany (*n* = 62), together with their core-genome based high relatedness and possession of nearly identical VAG patterns indicates that wolves could indeed play a role as vector in the epidemiology of the recurrent HS outbreaks in Germany.

After more than 150 years, the first reproduction of free ranging wolves in Germany was recorded in the year 2000 in the federal state of Saxony. Starting from the Lusatian border region to Poland (regional cluster R2 of this study), wolves have now repopulated large parts of the North German Plain northeast of a line linking the cities of Dresden and Bremen, predominantly comprising the federal states of Brandenburg, Mecklenburg-Western Pomerania, Lower Saxony, Saxony, and Saxony-Anhalt [25] (Figure 1). Besides the main range described above, scattered territories have been established in the federal states of Baden-Wuerttemberg, Bavaria, Hesse, Thuringia, North Rhine-Westphalia, Rhineland-Palatinate, and Schleswig-Holstein so far. In 2018–2019, the annual German wolf monitoring counted 105 packs, 40 couples and 12 resident single animals [25]. Occupation of territories is an ongoing and very dynamic process, resulting in a continuous rise in the wolf population. According to recent studies considering the habitat suitability for wolves in Germany, there may be enough living space for 700 to 1400 packs [36]. Genetic studies found that both the wolves of western Poland and eastern Germany, currently termed the Central European Lowland population [37], originated from two forest areas located in the Olsztyn district in northeast Poland [38].

Comparison of wolf population dynamics and HS occurrence in Germany revealed a close spatiotemporal relationship. This was especially prominent for initial HS cases in well-separated geographical regions which occurred within one (R6, R8, R9), two (R1, R3), four (R7), and seven (R4) years after wolves became resident in close vicinity to the outbreak sites (Supplementary Figure S1). With respect to the official DBBW wolf monitoring data, we found that 25, 50, 75 and 90% of the 83 HS cases investigated occurred after wolves became resident in a territory 7, 10, 24 and 44 km away from the outbreak site, respectively. However, in region R5 (Figure 1), harbouring one of the largest contiguous forest areas in Germany, HS initially occurred in November 2014, but the appearance of a resident wolf couple was not officially proven until the monitoring year 2017–2018. Nevertheless, a press release about a free ranging wolf in that area was already given in 2007 [39], and in April 2014 a young male died due to roadkill [25]. Thus, it appears highly likely that at least dispersing animals were present in R5 even before 2017. Based on this supposition, the linear distance of 25, 50, 75 and 90% of the HS outbreak sites to the centre of the closest wolf territory would be 7, 10, 20 and 28 km, respectively, and only three outbreaks would be more than 40 km away. This corresponds well with the sizes of German wolf territories which encompass between 100 and 350 km^2^, the mean home range size calculated from twelve packs in Lusatia was 215 km^2^ [25,40]. Nevertheless, a study monitoring a pack in Mecklenburg-Western Pomerania found a general home range covering 500 km^2^ [41]. Assuming a circular shape of these territories, their diameter would vary between 11 and 25 km. Actually, the ranges are neither fixed in size nor regular in shape but are influenced by different factors, e.g., types of habitat, human population density, road density, abundance of prey species (notably roe deer but also red deer and European wild boar), season and also neighbouring wolf territories [40,42,43,44,45]. Home range size also depends on age, as subadult animals might take long excursions out of the territory of their pack [45]. In a telemetry study of six GPS-GSM collared wolves (two adults, four juveniles) the calculated average and maximum walking distances per day ranged from 7.8 to 13.4 km and from 24.2 to 68.1 km, respectively [45]. It is noteworthy that dispersing wolves might travel very long linear distances up to 800 km to establish their own territories [45,46].

However, a HS outbreak (isolate IHIT37581) 84 km away from the nearest wolf territory located in R4 occurred in February 2014 on a pig farm in the state of Mecklenburg-Western Pomerania. As this farm purchased piglets from a free-range pig breeding farm in the R3 area of Lower Saxony (Bodo Thom, personal communication) introduction of *Pm*B might be due to animal trade. This is further supported by phylogenetic comparison as isolate IHIT37581 was found genetically indistinguishable from isolates of regions R1, R2 and R3 but distant from those of R4 (Figure 3). Interestingly, the isolate IHIT37582 recovered in 2016 from another pig farm in Mecklenburg-Western Pomerania, 45 km from the closest wolf territory at that time, was genetically identical to isolate IHIT37581. Consultation with the affected farmer revealed that he obtained pigs from the same breeder in R3. Furthermore, another two isolates from geographically separated outbreaks in domestic pigs (IHIT37579, 2018; IHIT37580, 2017) in Mecklenburg-Western Pomerania were genetically indistinguishable from each other but also from isolates from wild and domestic animals (cattle, pig) of the R4 region, which is approx. 120 and 220 km away from the outbreak sites, respectively. These cases indicate that in domestic pigs, which are commonly kept under high biosecurity measures, animal trade might play an important role in HS epidemiology. 

In the core genome-derived phylogenetic tree (Figure 3) the isolates IHIT36478 and IHIT36485 from wolves GW858m and GW809f, respectively, clustered best with HS outbreak isolates from the same region (R1 and R3, respectively). In contrast, isolate IHIT36466 from the juvenile wolf GW840m, which died in a road accident in the West of Germany, showed highest phylogenetic relatedness to HS outbreak isolates from the R4 area in the East. Indeed, genetic studies carried out within the framework of the German wolf monitoring revealed that the father (GW651m) of the killed animal originated from the Ueckermünde pack (R4), migrating more than 390 km westbound [47]. These findings open a new perspective on the epidemiology of HS. To date, only ungulates were known to harbour the infectious agent and particularly inapparently infected carrier animals, shedding the bacteria with their nasal excretions, are assumed to play a major role for maintaining the epidemiological cycle [48]. However, taking inhalation or ingestion as the basis of natural infection, it remained unclear how carriers could transmit the large numbers of bacteria (10^7^ to 10^12^ cfu) necessary to initiate an infection [49]. On the other hand, experimental subcutaneous inoculation of just 10^4^ to 10^7^ cfu was proven to produce clinical disease consistently [19,48]. As reported for wolves hunting moose (*Alces alces*) on the Isle Royale, predation efficiency, i.e., ratio of moose tested and moose killed, was 7.8%, with another 9.1% animals just wounded [50]. Official data concerning livestock attacks in various German states [51,52] also show that wolves injure some prey without killing them. As described in 50 human wound infections resulting from dog bites, *P. multocida* was isolated from 24% of the cases [53]. As a consequence, it is likely that wolves harbouring HS-causing *Pm*B in their oral cavity will transfer it to susceptible prey species during bite attacks, thus setting wound infections, which can lead to septicaemia and characteristic signs of clinical HS. On the other hand, in at least two confirmed cases from Germany, wolves fed on *Pm*B-infected cattle carcasses, thus completing a possible epidemiological cycle for HS. 

For further investigations, it will be of great interest whether epidemiological links between existing wolf territories and HS cases also occur in other countries. With regard to our study, it would be of particular importance to compare the German *Pm*B isolates with the isolates obtained from HS outbreaks in northeast Poland between 1991 and 1995 [21], as that region is the origin of the German wolf population [38]. It is noteworthy that approx. one quarter (22.9%) of all HS cases in our study affected European wild boar, thus indicating a special role of this animal species in the epidemiology of the disease. European wild boar represents 17.7% of all biomass consumed by wolves in Germany [44]. Larger animals especially could survive initial wolf attacks, but later might succumb to HS because of *Pm*B bite wound infections. In contrast, as European wild boars feed on carcasses of, e.g., fallow and roe deer, an oral route of infection must also be taken into consideration. Furthermore, by sharing pastures and/or water sources, European wild boars might be an epidemiological link to HS in domestic animals.

## 5. Conclusions

With our study we have demonstrated for the first time that carnivores, i.e., wolves, might harbour *Pm*B as a component of their oral microbiota. Isolates from wolves are genetically very closely related to isolates from HS outbreaks in wild and domestic ungulates, which repeatedly emerged over the past decade in Germany. Furthermore, there is a strong indication that wolves can carry the pathogen over long distances. The close spatiotemporal relationship between established wolf territories and HS outbreaks among wild and domestic ungulates indicates a major role of wolves in the epidemiological cycle of HS in Germany. However, especially for domestic pig, animal trade must be considered as a possible route of disease transmission.

## Figures and Tables

**Table 1:** Distribution of virulence-associated genes among 103 *Pm*B-ST122 strains.

Gene Category and Name	*Pm*B Isolates Germany/Hungary (*n* = 68)	Global *Pm*B Strains (*n* = 35)
	Positive strains (%)	
Adhesins/colonization factors		
*ptfA*	100	100
*fimA*	100	97.1
*hsf-1*	0	0
*hsf-2*	100	97.1
*pfhB-igB*	100	94.3
*pfhB1*	0	0
*pfhB2*	100	100
*tad locus*	0	0
Toxin		
*toxA*	0	0
Iron regulation/acquisition		
*afuCBA*	100	100
*ccmABCDEF*	100	97.1
*exbBD-tonB*	100	97.1
*fecBCDE*	100	100
*fbpABC*	100	100
*fur*	100	100
*hgbA*	0	0
*hgbB*	100	94.3
*tbpA*	0	77.1
Extracellular enzymes		
*nanB*	0	2.9
*nanH*	100	97.1
*neuA*	100	100
*nanATEK*	100	100
*nanR*	0	0
*pm*HAS	0	0
*siaPT-nanM*	100	100
Oxidative stress		
*sodA*	100	97.1
*sodC*	100	97.1
Secretion systems		
T2SS (“DR93_1687”)	0	0
T2SS/T3SS (“DR93_1692”)	0	0
Outer membrane proteins/protectins		
*ompA*	100	97.1
*ompH*	100	97.1
*oma87*	100	97.1
*plpB*	100	100

## Data Availability

Annotated genome sequences of *Pasteurella multocida* isolates sequenced herein were deposited to the publicly available repository of the National Center for Biotechnology Information under Sequence Read Archive (SRA) numbers SRR13148914 – SRR13148986 and SRR13838340. GenBank accession numbers of other *P. multocida* genomes used in the study are listed in Supplementary Table S3.

## Supplementary Materials

The following are available online at https://www.mdpi.com/article/10.3390/microorganisms9091999/s1, Table S1: Outbreak sites of Haemorrhagic septicaemia in Germany and their linear distance to the assumed geographic centre of the closest wolf territory, Table S2: Whole genome sequence analysis data of *Pasteurella multocida* HS isolates from this study and of publicly available genomes, Table S3: Descriptive data of 150 wolves investigated for *Pasteurella multocida*, Table S4: Reference sequences and description of virulence-associated genes, capsular and lipopolysaccharide biosynthesis genes that were used for in silico screening of *P. multocida* genomes, Figure S1: Location of HS outbreak sites (red asterisks) in relation to proven wolf territories (grey dots) per monitoring year. Regional outbreak clusters R1–R9 are marked by black circles. In 2012–2013 no outbreaks were recorded. For 2019–2020 only outbreaks up to end of September were included.
