# Supplementary material for: Re-Emergence and Spread of Haemorrhagic Septicaemia in Germany: The Wolf as a Vector?
Source: Microorganisms. 2021 Sep 21;9(9):1999. doi: 10.3390/microorganisms9091999 (PMC8465458; doi:10.3390/microorganisms9091999)
Supplement: Supplementary file 1 [file microorganisms-09-01999-s001.zip › Supplementary Table S4.pdf]

Supplementary Table S4: Reference sequences and description of virulence-associated genes, capsular and lipopolysaccharide biosynthesis genes that were used for *in silico* screening of *P. multocida* ST122[RIRDC] genomes

Gene category and name	Description	GenBank accession no. of reference sequence	Reference strain
**Biosynthesis of capsule**			
hyaD-hyaC	Specific genes of capsular type A	CP003022.1:910400- 914575	*Pm* 36950
bcbD	Specific genes of capsular type B	AF169324.1:17690-16422	M-1404
dcbF	Specific genes of capsular type D	AF302465.1:2892-4397	P934
ecbJ	Specific genes of capsular type E	AF302466.1:3246-5363	P1234
fcbD	Specific genes of capsular type F	AY604234.1:1-3156	J-4103
**Biosynthesis of LPS**			
locus_tag "NCTC10382_00021“	LPS genotype 1 (carboxylate/amino acid/amine transporter)	LS483473.1:16183-17091	NCTC 10382
locus_tag "NCTC10323_00016“	LPS genotype 2 (Glycosyl transferase family 2)	LR134532.1:12164-13006	NCTC 10323
locus_tag "ATO47_06115"	LPS genotype 3 (beta-1,4 galactosyltransferase)	CP013291.1:1387020-1386238	ATO47_06115
latB	LPS genotype 4 (putative acyltransferase 3)	KM670447.1:3565-1715	P2192
rmlC	LPS genotype 5 (putative dTDP-4-dehydrorhamnose 3,5-epimerase)	JN571483.1:13180-13722	P2095
nctB	LPS genotype 6 (putative beta-1,3 N-acetyl-glucosyltransferase	KJ689443.1:4447-3443	P1573
ppgB	LPS genotype 7 (biosynthesis of 1[(4 aminobutyl)amino]-3-hydroxy-1-oxopropan-2-yl hydrogen phosphate"	JX987238.1:3739-4788	P1591
natF	LPS genotype 8 (predicted1,4 N acetylgalactosamine transferase)	KM670448.1:4920-5795	P2723
**Adhesins/Colonization factors**			
ptfA	Type 4 fimbriae	KP726888.1:1-435	P-1059 (ATCC 15742)
fimA	Fimbriae (from Pm70)	KP660167.1:1-1125	UNMSM
hsf-1	Autotransporter adhesion (from Pm70)	AE004439.1: 840331-832193	Pm70
hsf-2	Autotransporter adhesion (from Pm70)	AE004439.1:1775628-1779527	Pm70
pfhB-igB	Filamentous hemagglutinin and putative immunoglobulin binding protein	AY035342.1:9393-11444	232
pfhB1	Filamentous hemagglutinin (FhaB) protein	AE004439.1:64167-72014	Pm70

Gene category and name	Description	GenBank accession no. of reference sequence	Reference strain
pfhB2	Filamentous hemagglutinin (FhaB) protein	ALBZ01000007.1:1250-5997	P52VAC
tad locus (*flp1-flp2-tadV-rcpCAB-tadZABCDEFG*)	Non-specific tight adherence protein locus (colonization island)	CP003328.1:946117-957784	PmHB03
**Toxin**			
toxA	G-protein deamidating *P. multocida* toxin	AY864768.1:22-3879	*Pm* clone HN-13
**Iron regulation/acquisition**			
afuCBA	Afu iron transport system	AE004439.1:1124243-1130644	Pm70
ccmABCDEF	Proteins involved in posttranslational attachment of heme	AE004439.1:5567-10363	Pm70
exbB	Accessory protein Ton-dependent transport of iron compound	AF468825.1:531-989	PM25
exbD	Accessory protein Ton-dependent transport of iron compound	AF468825.1:998-1387	PM25
tonB	Proteins forming the TonB-dependent transporter	AF468825.1:1384-2154	PM25
fecB	Iron-dicitrate transporter substrate-binding subunit	CP014618.1:1113112-1114008	Pm-3
fecC	Iron-dicitrate transporter permease subunit	CP014618.1:1114008-1114991	Pm-3
fecD	Iron-dicitrate transporter subunit FecD	CP014618.1:1114991-1115983	Pm-3
fecE	Ferric citrate ABC transporter ATP-binding protein	CP014618.1:1115983-1116756	Pm-3
fbpABC	Ferric binding proteins	AE004439.1:52929-56773	Pm70
fur	Ferric uptake regulation protein	KP659942.1:1-441	UNMSM
hgbA	Putative TonB-dependent receptor	AF237932.1:1-2904	Not specified
hgbB	Putative hemoglobin-iron uptake protein	KX832982.1:1-755	Pm04
tbpA	Transferrin-binding protein A	DQ782801.1:1-2334	Pm D:1
**Extracellular enzymes**			
nanB	Sialidase NanB	AF274868.1:77-3289	86-1913
nanH	Sialidase NanH	AF274869.1:90-2333	86-1913
neuA	CMP-sialic acid synthetase	AE004439.1:209243-209914	Pm70
nanATEK	Structural proteins involved in sialic acid catabolism	CP008918.1:655947-659112	ATCC 43137
nanR	Regulatory protein involved in sialic acid catabolism	UGSY01000001.1:313035-314003	*P. testudinis* NCTX 12150
*pm*HAS	Hyaluronan synthase	AF036004.2:1-2919	P-1059 (ATCC 15742)

Gene category and name	Description	GenBank accession no. of reference sequence	Reference strain
siaPT-nanM	Proteins that form the TRAP system required for sialic acid uptake; NanM: N-acetylneuraminate epimerase	CP008918.1:660846-665052	ATCC 43137
**Oxidative stress**			
sodA	Superoxide dismutase A	AE004439.1:185-829	Pm70
sodC	Superoxide dismutase C	AE004439.1:2190308-2190868	Pm70
**Secretion systems**			
locus_tag "DR93_1687“	Type II secretion system	NZ_CP008918.1:1761952-1761098	ATCC 43137
locus_tag "DR93_1692"	Type II and type III secretion system	NZ_CP008918.1:1767124-1765769	ATCC 43137
**Outer membrane proteins/protectins**			
ompA	Outer membrane protein A	KU342632.1:1-1077	768
ompH	Outer membrane protein H	JX473022.1:1-1023	XJNKY-12-YF1
oma87	Outer membrane protein 87	U60439.1:345-2714	PBA100
plpB	Pasteurella lipoprotein B	KP659994.1:1-831	UNMSM
