# Supplementary figures and images for: Re-Emergence and Spread of Haemorrhagic Septicaemia in Germany: The Wolf as a Vector?
Source: Microorganisms. 2021 Sep 21;9(9):1999. doi: 10.3390/microorganisms9091999 (PMC8465458; doi:10.3390/microorganisms9091999)

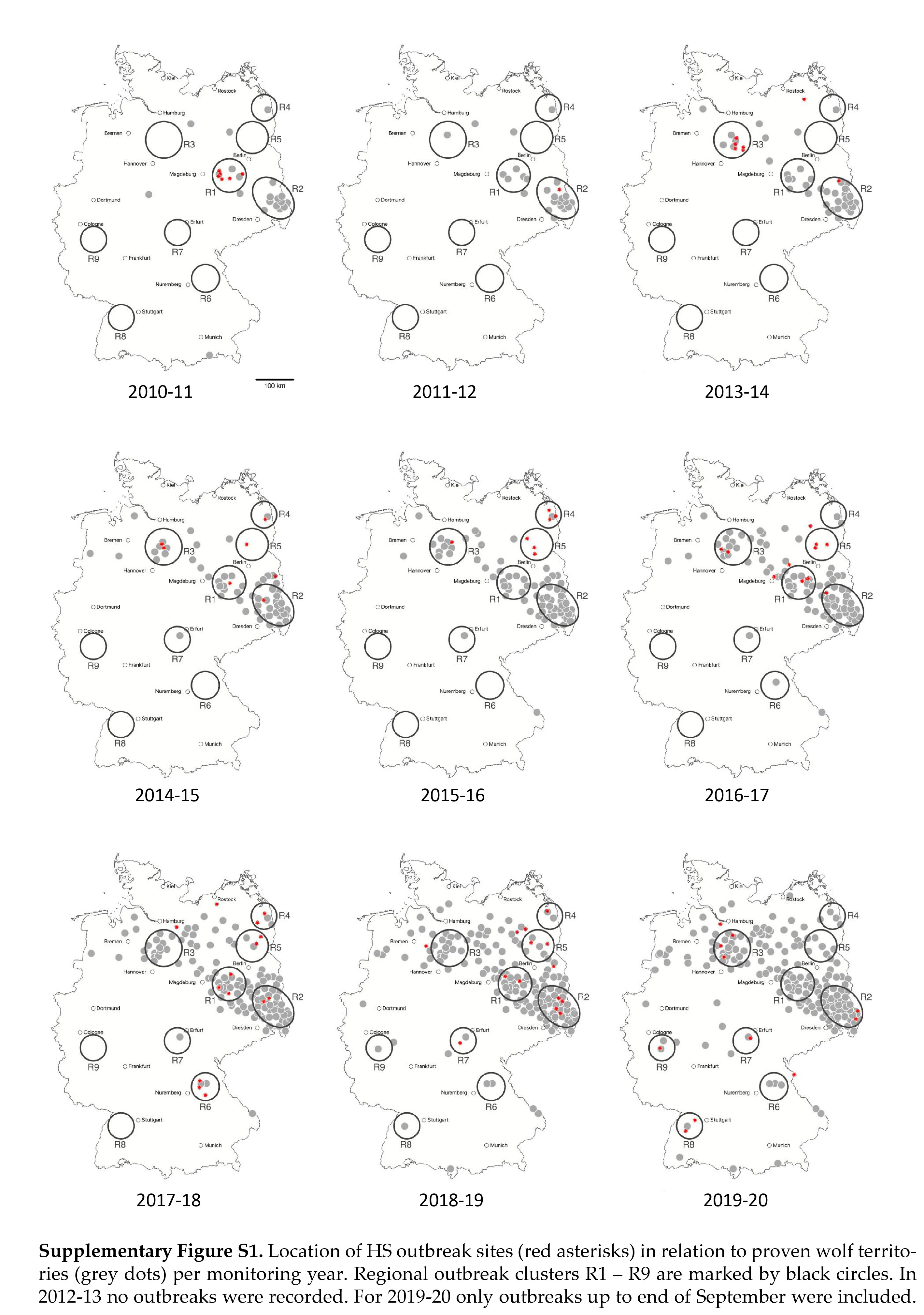

Supplement: Supplementary file 1 [file microorganisms-09-01999-s001.zip › Supplementary Figure S1.jpg]
